# Opossums. An adaptive radiation of New World marsupials, by Robert S. Voss & Sharon A. Jansa: Johns Hopkins University Press, 2021, pp. x + 313, Hardcover ISBN: 9781421439785

**DOI:** 10.1007/s10329-021-00931-9

**Published:** 2021-07-07

**Authors:** Eckhard W. Heymann

**Affiliations:** Verhaltensökologie & Soziobiologie, Deutsches Primatenzentrum - Leibniz-Institut für Primatenforschung, Kellnerweg 4, 37077 Göttingen, Germany

Why should a primatological journal publish a review of a book on opossums, animals that as marsupials (mammalian infraclass Metatheria) are phylogenetically quite remote from the primates (infraclass Eutheria)? A simple answer could be that opossums, or more specifically woolly opossums of the genus *Caluromys*, have been employed as a model for the early evolution of primates, particularly of foraging adaptations and locomotion (Rasmussen 1990; Lemelin 1999; Schmitt and Lemelin 2002). But there is more to learn from opossums than just from *Caluromys*. Extant opossums (Didelphiomorphia: Didelphidae) represent a speciose (116 species recognized in this book), diverse and ecologically very interesting family that is mainly distributed in the Neotropics, but also extends into non-tropical regions of the Americas. They reach their highest diversity in lowland tropical rainforests. Thus, opossum distribution and habitats largely overlap with Neotropical primates, making it likely that they have at least some indirect ecological interactions. And more generally, they show some interesting similarities, but also differences with primates, particularly strepsirrhines. Charles-Dominique (1983) had already suggested that opossums and African strepsirrhines occupy similar ecological niches. His comparison of opossums and African strepsirrhines resulted in questioning a link between ecological and social adaptations, a link that lies at the heart of the socio-ecological model which is so widely applied in primatology (Janson 1999). Thus, opossums can (and should) be of much broader interest to primatologists. I frankly admit that my conception of these creatures was rather rudimentary before reading this book, and I only learned now how speciose, diverse and ecologically interesting they are.

**Fig. 1:**
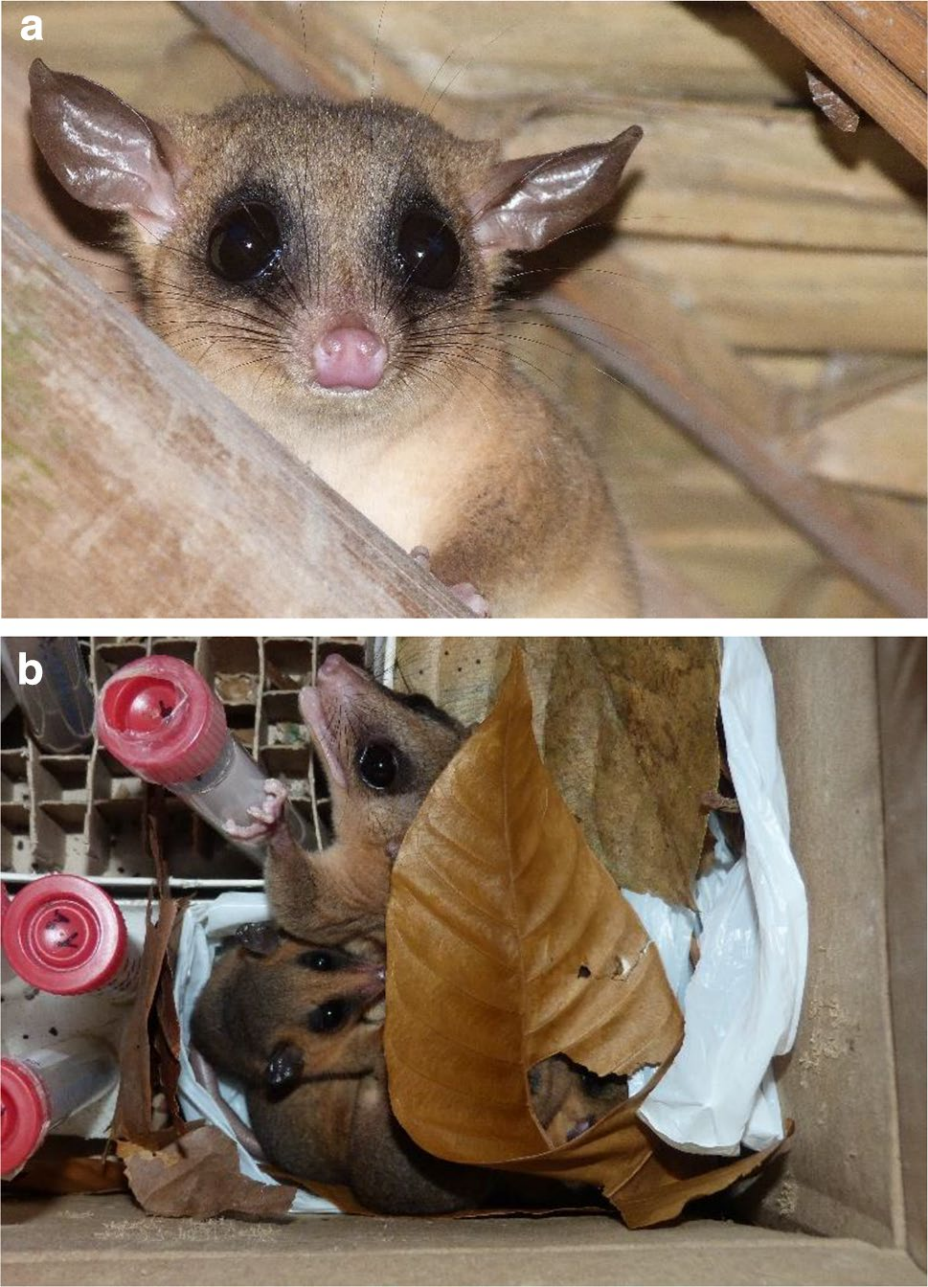
**a** A *Marmosa*, probably *M. constantiae*, under the palm-thatched roof of a building at Estación Biológica Quebrada Blanco, northeastern Peruvian Amazonia. **b** Probably the same adult individual with its young, nesting in a cardbox

Since opossums are mainly nocturnal, Neotropical primatologists will rarely encounter them during field work, unless they live in the camp or field station, or use equipment for nesting (Fig. 1). Neotropical primates will very rarely interact with them directly, perhaps except for night monkeys. However, since the diet of those species for which information is available is largely based on invertebrates, small vertebrates and fruits, there is quite likely some overlap with primate diets, particularly with those of the frugivorous–faunivorous callitrichids and cebids. As becomes clear from the presentation of opossum diets (Chapter 9), an obvious constraint for a better understanding of their feeding ecology and for comparisons with other taxa is the difficulty of observing opossums directly; most dietary information is based on the examination of faeces and stomach contents, both being biased against more easily digestible material. Metabarcoding, which is increasingly used in primate ecology (Rowe et al. 2021; Mallott et al. 2015) but does not yet seem to have been used in opossum studies, will render new insights into the synecology of opossums and primates. At this point, one appreciates how privileged we are as primatologists by generally being able to watch and follow our study subjects and collect ecological and behavioural data directly, albeit not on all species.

Interest in opossums should not be restricted to Neotropical primatologists. Rather, scholars working on strepsirrhines will also benefit from reading this book. Not only do some opossums and strepsirrhines show phenotypic similarity (compare Fig. 1 with photos of mouse lemurs and galagos), but there are other similarities (but also differences) that make a comparative look worthwhile. Opossums and strepsirrhines overlap strongly in the range of body mass, but the degree of sexual dimorphism is much stronger in most opossums compared to strepsirrhines (compare Table 5.1 in this book with Table 1 in Kappeler 1991). Like many nocturnal lemurs, lorises and galagos, opossums are solitary foragers, possibly occupying similar ecological niches (Charles-Dominique 1983). But while long-term studies of strepsirrhines revealed that despite solitary foraging, social organization and structure can be quite varied and more complex than the term “solitary foragers” insinuates (Kappeler 2012; Kappeler et al. 2017), current evidence for opossums seems to indicate that they have relatively simple social systems, with social interactions between adults being largely restricted to mating and some agonism, mainly over food (Chapter 7: Behavior). It would not be surprising if long-term studies of individually known opossums revealed more complexity than currently acknowledged. Differences in the degree of home-range overlap (Chapter 13: Population biology) and the variable degree of sexual size dimorphism nourish such a conjecture. But even if long-term studies confirmed their highly solitary nature, an interesting question emerges: what constrains opossums from developing more complex social systems? Is it brain size, cognition, the short life span or other aspects of opossum life history, e.g., their reproductive biology? These are just a few thoughts that came to my mind while reading this book; there are many more places in it that stimulate questions and ideas.

The book is organized into five sections (phylogenetic context and historical biogeography; opossum classification and diversity; opossum phenotypes; opossum natural history; synthesis) which comprise the 14 chapters. Each chapter ends with a discussion that concisely and critically evaluates the data and information provided in the chapter and identifies open questions. As the authors note in the introduction, the book does not attempt to comprehensively review the literature on opossums. Nevertheless, it is a very rich source of information; one can be content with this information, or use it as a starting point to delve deeper into opossum biology. The 26 photos, although only b/w, provide a good idea of the phenotypic variation within the Didelphidae. They also illustrate fascinating aspects of opossum natural history; e.g., Fig. 4.12 showing a *Philander melanurus* attacking a venomous snake or Fig. 7.2 showing a female *Marmosa murina* carrying two rather large infants (which reminded me of heavily burdened infant carriers in tamarins and marmosets). Another highly interesting aspect of opossum natural history is the resistance to snake venoms and poisonous amphibian skin secretions (Chapter 6: Physiology). I wondered whether anything is known about such resistance in tarsiers that prey on venomous snakes (Niemitz 1984). I am only aware of hints to reduced susceptibility to α-neurotoxins in catarrhines, but this perhaps evolved as a response to predation by rather than upon snakes (Harris et al. 2021).

In conclusion, I highly recommend this book to primatologists, particularly to those working with Neotropical primates and with strepsirrhines. All information is presented in a highly accessible way and provides a lot of food for thought. I am convinced that everybody who shared my ignorance of opossums before reading this book will also share my enthusiasm after reading it, and will benefit from thinking more—or starting to think—about similarities and differences between opossums and primates.
